# The effect of green tea consumption on oxidative stress markers and cognitive function in patients with Alzheimer’s disease: A prospective intervention study

**Published:** 2016

**Authors:** Horrolein Arab, Soleiman Mahjoub, Karimollah Hajian-Tilaki, Mehdi Moghadasi

**Affiliations:** 1 Student Research Committee, Babol University of Medical Sciences, Babol, Iran.; 2 Cellular and Molecular Biology Research Center, Health Research Institute, Babol University of Medical Sciences, Babol, Iran.; 3 Based Health Products Research Center, Babol University of Medical Sciences, Babol, Iran.; 4 Department of Social Medicine and Health, Babol University of Medical Sciences, Babol, Iran.; 5 Department of Neurology, Mehr-Avaran-Shomal Nursing Home, Sari, Iran.

**Keywords:** Alzheimer’s disease, Antioxidants, Green tea, Oxidative stress, Cognitive function.

## Abstract

**Background:**

Alzheimer’s disease (AD) is the most prevalent degenerative disorder of the brain among elderly individuals. Many studies indicate that oxidative stress is an important pathogenic factor which involves oxidizing macromolecules such as DNA, lipids, and proteins in AD. Green tea is a rich source of antioxidant compounds that can remove radical oxygen species. The purpose of this study was to investigate the influence of green tea consumption on markers of oxidative stress in AD.

**Methods:**

In this prospective intervention study, 30 patients with severe AD were recruited. The diagnosis of AD was made based on the National Institute of Neurological and Communicative Disorders and Stroke and the Alzheimer’s disease and Related Disorders Association (NINCDS/ADRDA) criteria. Brain magnetic resonance imaging (MRI) and computed tomography (CT) scan as well as Mini-Mental State Examination (MMSE) were performed for all participants in which four green tea pills were administered daily for 2 months (2 g/day in 2 divided doses). The plasma total antioxidant capacity, 8-hydroxy-2’-deoxyguanosine levels (8-OHdG), malondialdehyde (MDA), carbonyl content, and MMSE scores were measured at baseline and at the end of the study period.

**Results:**

The levels of MDA, 8-OHdG and carbonyl decreased significantly as compared to baseline values (P=0.002, P=0.001 and P=0.037, respectively). Whereas, the total antioxidant capacity of plasma and MMSE scores significantly increased at end point (P=0.000, P=0.043, respectively).

**Conclusion:**

The findings indicate that consumption of green tea for two months by with the improvement of antioxidant system exerts beneficial effect on cognitive function.

Alzheimer’s disease (AD) is a progressive, irreversible neurodegenerative disorder of the brain that occurs in the elderly individuals. AD is associated with cognitive and functional impairments (1). The definitive diagnosis of AD is possible only by histologic examination(2) but based on clinical and imaging technics such as computerized tomography (CT) scans, magnetic resonance imaging (MRI),80-95% of cases can be correctly recognized (3). AD is the most common cause of dementia, which affects 35 million people in the world. Age is the most important determinant of the disease and the risk of AD doubles every 5 years after 65 years (4).

Recent studies have indicated that oxidative stress is an important pathogenic factor in different diseases including AD, which occurs by oxidizing macromolecules such as DNA, lipids, and proteins (5-8). The results of earlier studies regarding neuroprotective effects of antioxidants are controversial (9). 

However, the latest studies have shown that antioxidants have beneficial effects in patients with AD and reduce the progression of the disease (10). Green tea is highly popular worldwide because it is a safe and non-toxic beverage that has no side-effects. Green tea is one of the most favorite beverages around the world, and is now known as medicinal plant; therefore its therapeutic properties have been studied extensively (11, 12). Green tea is produced from the leaf of *Camellia Sinensis,* of the *Theaceae* family (13). 

Polyphenolic compounds with high antioxidant capacities called catechins are present in large quantities in green tea (14), and their anti-aging (15), anti-stroke (16), anti-cancer (17, 18), and anti-diabetic (19, 20) effects have been shown in various studies. Nonetheless, the effect of green tea on oxidative stress markers in AD requires further studies. This study was performed to investigate the influence of green tea on oxidative stress and cognitive function in patients with AD.

## Methods

**Participants:** In this prospective intervention study, we enrolled thirty patients with severe AD at the Mehr-Avaran-Shomal Nursing Home in Sari, northern Iran. The study protocol was approved by the Ethics Committee of Babol University (No. 3609, approved on March 19, 2013). This study was registered in Iranian Registry of Clinical Trials (IRCT201402233684N5). Written informed consent was obtained from the caregiver or the patient’s legal representative prior to the initiation of the study procedures, as the patients could not provide informed consent. The patient participants of this study were the same population of our previous research (21). 

The diagnosis of Alzheimer’s disease was confirmed based on the National Institute of Neurological and Communicative Disorders and Stroke and the Alzheimer’s disease and Related Disorders Association (NINCDS/ADRDA) criteria. Data in regard to Mini-Mental State Examination (MMSE) scores (0–10), brain MRI, and CT scanning were provided based of clinical examination, interview and the patient’s medical records. Other causes of dementia were excluded by appropriate clinical examination and imaging procedures (MRI and CT scan) and laboratory (TSH, T3, T4, CBC/DIFF, BUN/CR, FBS, LFT, and ESR) tests. Exclusion criteria were (a) green tea allergy; (b) inflammatory and infectious diseases such as hepatitis, anemia, and diabetes; and (c) consumption of supplements with antioxidant effects such as vitamins A, C, E and folic acid. All patients were treated with cholinesterase inhibitors (donepezil and memantine) for at least 6 months before entry to trial.

**Laboratory methods:** Patients received 4 green tea pills a day in two divided doses for two months. Each pill of green tea leaf powder (500 mg) contained 50 mg of total polyphenols, including epigallocatechin gallate (EGCG), epicatechin (EC), epigallocatechin (EGC), and epicatechin gallate (ECG). We provided green tea pills with IRC-1228144011 from a group focusing on hygiene and safety for the food and pharmaceutical industries (Dineh IRAN Co., Qazvin, Iran). 

The consumption of other supplements that could have antioxidant effects was forbidden during the intervention and for a 7-day washout period before starting intervention. The caregiving staff recorded any changes in the health status of subjects or consumption of medication, as well as occurrences of any side effects. No patients dropped out of the study due to inconvenience or adverse effects related to the treatment. For those who were unable to swallow the pills, the pills were crushed in a mortar before administration. Venous blood samples were collected in sodium heparin tubes before and after the dietary intervention from each subject under fasting conditions. Plasma was isolated by centrifugation at 1000 rpm for 10 min and aliquots were kept at −80°C until analysis. All tests were analyzed at baseline and again after 2 months of green tea pill consumption.

**TBARS assay:** MDA as a biomarker of lipid oxidation was determined by the thiobarbituric acid reactive substance (TBARS) test. The reaction between MDA and thiobarbituric acid (TBA) produced a colored compound that absorbs at 535 nm. The results of MDA concentration were expressed as nmol/ml (22).

**Protein carbonyl assay**: Protein carbonyl content is widely known as a marker of protein oxidation. There are various methodologies for the quantification of protein carbonyl content; in all samples we used an assay developed by Levine et al. to evaluate protein carbonyl levels. According to this assay, 2,4-dinitrophenylhidrazine (DNPH) and the carbonyls react and generate a stable product, 2,4-dinitrophenyl hydrazone, that can be verified by a spectrophotometer at 370 nm (23). The total protein amount due to reductions in the duration of the washing step was evaluated in the last step. Sample proteins were determined by a commercial kit of total proteins (ZIESTCHEM CO., Tehran, Iran). Carbonyl content was expressed as nmol/mg protein (24).

**FRAP assay:** Total antioxidant capacity considers the cumulative actions of all antioxidants available in plasma and body fluids. Plasma total antioxidants were measured by the ferric reducing ability of plasma (FRAP) assay. In the FRAP assay, antioxidants in the plasma convert the ferric tripyridyltriazine (FeIII-TPTZ) complex to a blue colored ferrous (FeII) form with absorbance at 593 nm FeSo_4_ that was used as a standard. Evaluations were expressed as µmol/l (25). 

**8-OHdG assay:** 8-OHdG, a DNA base-modified product, is one of the most widely used markers within cells for the measurement of oxidative DNA damage (26). The concentrations of 8-OHdG were determined by sandwich enzyme-linked immunosorbent assay (ELISA) using a commercial kit (Chongqing Biospes Co., Ltd). According to the kit method, a plate was coated with purified anti-8-OHdG antibody, the sample was added, and any 8-OHdG in the sample connected to the anti-8-OHdG antibody. Horseradish peroxidase (HRP) conjugated with anti-8-OHdG antibody as the detection of the antibody was added, and connected to the 8-OHdG antibody complex. Then, 3,3’,5,5’-tetramethylbenzidine (TMB) as a substrate was added and converted by the HRP to a blue product that changed to a yellow product after adding an acid stop solution. The absorbance of the microplate was recorded at 450 nm. The concentration of 8-OHdG was calculated, then, reported in ng/ml.

**Cognitive function assessment:** Cognitive function was assessed using the Persian language version of the MMSE which has a sensitivity of 90% and a specificity of 93.5% (27). The MMSE or Folstein test includes 30 questions regarding time and place, calculation, attention, language, and visual ability. This screening test was created for use in a clinical setting and is used considerably in epidemiologic research to estimate the severity of cognitive impairment at a specific time, and as a follow-up measure of cognitive impairment within a limited time. Scores from 0 to 30 are calculated (28). Higher MMSE scores indicate higher cognitive function, and 30 points is the maximum score. The score describes different levels of cognitive impairment: no cognitive impairment=25–30; mild cognitive impairment= 21–25; moderate cognitive impairment=11–20; and severe cognitive impairment=0–10 (29). The test was administered by a psychiatrist in the nursing home.

**Statistical analysis:** Data analysis was performed using the SPSS version 16.0 package. The significance level was determined as less than a P-value of 0.05. Paired sample t-test was used for comparison.

## Results

The demographic and medical features of the study participants are shown in table 1. Thirty patients (8 men and 22 women) with AD were enrolled into the study. The mean MMSE score ± SEM was 3.7±0.8 (range 0–10) at baseline. In table 2, the plasma oxidative stress markers and MMSE scores of the subjects at the beginning and the end of the trial are summarized. Consumption of green tea pills for two months significantly increased the total antioxidant capacity of plasma levels (from 1140.7±69.0 to 1391.1±54.9, P=0.000). 

The plasma levels of other markers significantly decreased, including 8-OHdG (from 957.0±52.5 to 719.7±39.6, p=0.001), MDA (from 4.3±0.7 to 2.4±0.3, P=0.002), and carbonyl content (from 1.9±0.4 to 1.2±0.3, P=0.037). In addition, MMSE scores significantly improved (3.7±0.8 to 3.8±0.9, P=0.043) after the green tea pills were administered.

**Table 1:** Demographic and medical features of the study participants

30 81±8.2 (67-91)	Number of subjects Age(year) Mean±SD (range)
	Sex, n (%)
8 (26.7) 22 (73.3)	Men Women
3.66±0.77	MMSE Score Mean(±SEM)

Abbreviations: MMSE, Mini-Mental State Examination

**Table 2:** Mean (±SEM) of oxidative stress markers and MMSE score of patients with Alzheimer’s disease before and after a two-month consumption of green tea pills

**Parameters**	**Status**	**No**	**Mean±SEM**	**P-value**
Total antioxidant capacity (µmol/L)	before after	30 30	1140.7±69.0 1391.1±54.9	0.000
8-OHdG (ng/mL)	before after	30 30	957.0±52.4 719.8±39.7	0.001
Malondialdehyde (nmol/mL)	before after	30 30	4.3±0.7 2.4±0.3	0.002
Carbonyl content(nmol/mg pr)	before after	30 30	1.9±0.4 1.2±0.3	0.037
MMSE Score	before after	30 30	3.7±0.8 3.8±0.9	0.043

Abbreviations: 8-OHdG8-hydroxy-2'-deoxyguanosine; MMSE, Mini-Mental State Examination

## Discussion

In this prospective intervention study, the antioxidative effects of green tea on oxidative stress and cognitive function were investigated using blood biochemical markers in patients with AD. The results of this study showed that green tea consumption substantially decreased MDA concentration, 8-OHdG concentration, and carbonyl content concentration. Additionally, green tea substantially increased the total antioxidant capacity of plasma concentrations and MMSE scores. These results are consistent with the hypothesis that antioxidants can reduce markers of oxidative damage. Several possible mechanisms of actions have been proposed for the antioxidative action of green tea. It is possible that green tea prevents iron-induced lipid peroxidation by chelating iron. Green tea catechins contain well-established metal-chelating structures. The dihydroxyl groups in the B ring and the gallate group at position 3 in the C ring may neutralize ferric iron to form redox-inactive iron, thereby protecting cells against oxidative damage. In addition, studies have shown that lipid peroxidation is a chain reaction. Therefore, the scavenging property of polyphenols may decrease the concentration of hydroxyl radicals and lipid free radicals, thereby terminating the initiation and extension of lipid peroxidation. 

A similar effect was also reported in the brain, in which the nervous tissue is susceptible to lipid peroxidation due to high levels of oxygen consumption and high phospholipids contents with polyunsaturated fatty acids, which are highly vulnerable to peroxidation. Flavonoids also reduce lipid peroxidation by restricting the entry of oxidants into the bilayer and the reproduction of lipid oxidation in the hydrophobic membrane matrix. The hypothesis has been investigated that flavonoids have the capacity to interact with membrane lipids, particularly with their polar head groups. The implications of these interactions contain the possibility of protection of lipid membranes from oxidation and other insults that could imperil membrane integrity and function (30). Reduced lipid peroxidation and MDA levels following consumption of green tea indicates a powerful antioxidant activity from green tea consumption, associated with the prevention of oxidative stress in patients with AD.

According to our results, the mean level of 8-OHdG concentrations as an indicator of DNA oxidation was significantly reduced after two months of green tea consumption. This reduction agrees with in vitro observations that have reported that green tea extracts lessened DNA damage by scavenging free radicals, boosting antioxidant abilities, and inhibiting β-amyloid (Aβ) (31, 32). Green tea can protect DNA from oxidation by inhibiting xanthine oxidase, which causes catabolism of purines and produces uric acid and ROS, and by increasing the levels of cytoskeletal and structural proteins, such as stabilizer proteins involved in chromatin organization, along with DNA, histone H1e, and H2b, which have been demonstrated to play crucial roles in protecting DNA (33, 34). Furthermore, green tea polyphenols suppress lipid peroxidation by exerting their antioxidative action via chelating metal ions, such as iron (Fe2+) and copper (Cu2+), and by preventing the generation of hydroxyl radical via the Fenton reaction, and protect DNA. These compounds may also shift an electron to ROS-induced radical sites on DNA and thereby prevent oxidative DNA alteration (35). In this research, we also observed slight meaningful diminutions in the mean level of carbonyl content as good indicators of oxidized proteins after two months of green tea consumption. Evidence suggests that phenolic compounds of green tea can act as antioxidants by decelerating protein oxidation reactions or by binding to proteins. The antioxidative mechanism in phenol-protein accumulations may be due to the ability of phenolic compounds to move oxidative damage from one phenolic site to the other, defending proteins from oxidation (36). Antioxidant activity in the serum reduces during aging (37). Several epidemiological and experimental investigations illustrated that increasing the total antioxidant capacity of plasma concentrations correlated with green tea consumption (38, 39). The results that we have obtained agree with earlier findings of increased total antioxidant capacities of plasma levels after green tea consumption. Most likely, green tea catechins perform antioxidant activities instead of other antioxidants after entering the blood plasma, and thereby increase the antioxidant activities. (40). They act as radical scavengers by donating electrons or hydrogen atoms from the hydroxyl moieties to the free radicals, and exert indirect antioxidant effects through the activation of transcription factors and antioxidant enzymes (41). 

Green tea polyphenols upregulate antioxidant protective enzymes, including superoxide dismutase and catalase, and elevate the activity of these two major oxygen radical species–metabolizing enzymes (35). Moreover, in peripheral tissue, it has been found that flavonoids and phenolic antioxidants at low concentrations activate the expression of some stress response genes, such as phase II drug-metabolizing enzymes, glutathione-s-transferase, and heme oxygenase-1 (42). Several studies illustrate that both the increased oxidative stress and decreased antioxidant defense system in brain neuronal cells are the major factors in the decline of cognitive function with age. Experimental studies showed both the decline of memory performance with age and decreased levels of dietary antioxidants. Our findings showed that MMSE scores improved slightly. It seems that dietary antioxidants may protect against oxidative damage in neuronal tissue and may impede degeneration of the neuronal system with aging. More recently, it has been inferred that polyphenol effects on the brain are mediated by the protection of neurons, enhancement of neuronal function and growth, and the effects on the cerebrovascular system, such as extended blood flow in the brain. It appears that polyphenolic flavonoids may exert neuroprotective actions by interactions with long-term potentiation proteins central to intracellular signaling cascades, which are crucial for neuronal survival, such as protein kinase and lipid kinase, which are responsible for neuroinflammation. In addition, several studies have contended that neuroprotective effects of green tea are not only due to those properties, but also due to acetyl cholinesterase inhibition, which may increase cognition in conditions related to cholinergic deficits (e.g., AD), inducing specific protein synthesis in neurons which could have a direct effect on cognitive functions, especially memory (43), increasing neuronal connection efficiency and dendrite morphology, enhancing synaptic plasticity via changing in the genetic expression of neurons (44), and modulation of serotoninergic, dopaminergic, and GABAergic neurotransmission (45-47). The limitation of this study is of its non-blinded, non-placebo controlled design. In the next project, the study design should be changed; a blinded, placebo controlled design would be appropriate to determine the benefits of green tea consumption. However, using green tea pills (each 500 mg) with exact amounts of total polyphenols (50 mg) and also investigation of DNA, lipid and protein oxidative markers with evaluation of total antioxidant capacity in the patient are the advantages of our study. In conclusion, our study suggests that consumption of green tea (2 g/day) increases antioxidant capabilities and reduces lipid peroxidation, DNA, and protein oxidation, and improves cognitive function in individuals with AD. In comparison to medical care and medication treatments, nutritional interventions can be more viable and cost-effective for managing AD.

## Notes

**Funding: **This study was funded by a grant from the Vice- Chancellery for Research of Babol University of Medical Sciences (Grant number: 9237016) and registered as MSc thesis of Horrolein Arab of the Faculty of Medicine (No: 52).

## Citation:

Arab H, Mahjoub S, Hajian-Tilaki K, Moghadasi M. The effect of green tea consumption on oxidative stress markers and cognitive function in patients with Alzheimer’s disease: A prospective intervention study. Caspian J Intern Med 2016; 7(3): 188-194.
